# Neural Repetition Effects in the Medial Temporal Lobe Complex are Modulated by Previous Encoding Experience

**DOI:** 10.1371/journal.pone.0040870

**Published:** 2012-07-19

**Authors:** Ciara M. Greene, David Soto

**Affiliations:** Division of Brain Sciences, Faculty of Medicine, Imperial College London, London, United Kingdom; University of Cambridge, United Kingdom

## Abstract

It remains an intriguing question why the medial temporal lobe (MTL) can display either attenuation or enhancement of neural activity following repetition of previously studied items. To isolate the role of encoding experience itself, we assessed neural repetition effects in the absence of any ongoing task demand or intentional orientation to retrieve. Experiment 1 showed that the hippocampus and surrounding MTL regions displayed neural repetition suppression (RS) upon repetition of past items that were merely attended during an earlier study phase but this was not the case following re-occurrence of items that had been encoded into working memory (WM). In this latter case a trend toward neural repetition enhancement (RE) was observed, though this was highly variable across individuals. Interestingly, participants with a higher degree of neural RE in the MTL complex displayed higher memory sensitivity in a later, surprise recognition test. Experiment 2 showed that massive exposure at encoding effected a change in the neural architecture supporting incidental repetition effects, with regions of the posterior parietal and ventral-frontal cortex in addition to the hippocampus displaying neural RE, while no neural RS was observed. The nature of encoding experience therefore modulates the expression of neural repetition effects in the MTL and the neocortex in the absence of memory goals.

## Introduction

The response of the brain to the re-occurrence of past information has been extensively investigated using tasks that require observers to retrieve and match details of past events against current perceptual input. Evidence from functional neuroimaging studies in human and single cell recording in animals has shown that the repetition of stimuli typically results in a reduction of neural activity, a phenomenon termed repetition suppression (RS) [1]. This process is thought to reflect more efficient processing in the brain and has been conceptually linked with behavioural priming mechanisms [2]. RS may be due to a sharpening of neural response [3], as previously processed information is represented by a more sparse configuration of neurons.

In memory paradigms that involve explicit retrieval of information, improved recognition performance has been associated with decreases in neural activity (i.e. RS) in regions of the medial temporal lobe (MTL) [4], [5], [6]. A pattern of increased activation (repetition enhancement, RE) is however sometimes seen [7], [8], [9]. The reasons behind this variation in the expression of neural repetition effects remain elusive. Recognition memory tasks typically require participants to make purposeful ‘old/new’ retrieval-based discriminations for test items that may or may not have been previously studied. As a result, neural repetition effects in putative memory substrates could be associated with multiple task-based constraints imposed by the memory test itself such as retrieval success and retrieval effort [10]. Furthermore, neural responses to the reappearance of old items during explicit recognition tests can be influenced by processes operating at encoding [11] and by the level of information processing during encoding [12], [13], even when initial encoding occcurs incidentally [14]. We hypothesised that the manifestation of repetition-related neural response may be dependent on the nature of previous encoding experience with the stimuli, and that this may be the case even in the absence of memory goals during the assessment of repetition effects.

**Figure 1:**
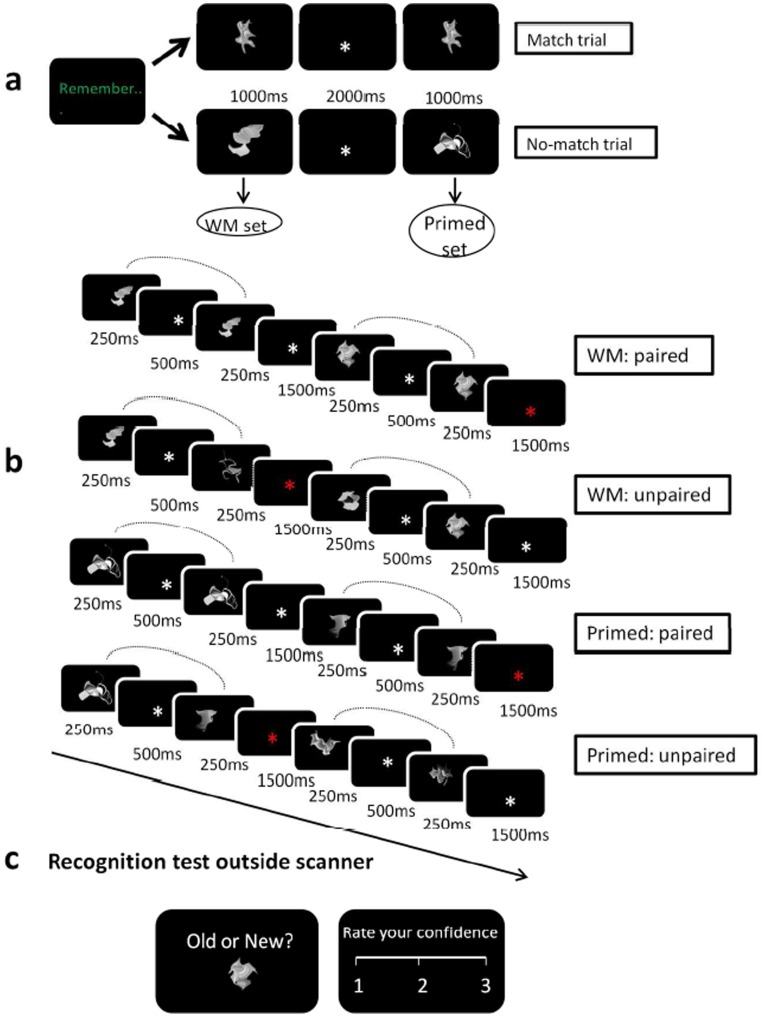
Experimental timeline. (A) Sample match and no-match trials from the WM phase. A memory cue and probe were presented for 1000 ms each, separated by a 2000 ms delay. (B) Sample trials from the repetition phase. Four repetition blocks were performed, corresponding to the four experimental conditions (WM set with paired repetition; WM set with unpaired repetition; Primed set with paired repetition; Primed set with unpaired repetition). The WM set was composed of memory cue items from the no-match trials in the study phase, while the memory probe items from no-match trials formed the Primed set. During the paired repetition blocks, items from either the WM or Primed set were briefly presented as repeated pairs, separated by a 500 ms interstimulus interval. During the unpaired blocks, the two stimuli presented within each trial were different items but were drawn from within the same (WM or primed) set. Each item was presented twice over the course of the block. To ensure attention to the display, participants responded with a button press when the fixation cross changed colour. (C) Sample trial from the memory recognition test performed outside the scanner. Participants indicated whether the item was old (previously seen) or new. They then rated their confidence in that judgement on a scale from 1 (not at all confident) to 3 (very confident).

Understanding how the nature of past encoding experience influences subsequent neural repetition effects is critical if we are to fully account for the implementation of episodic memory in the brain, but in order to isolate the role of encoding experience itself it is essential to minimize any contribution from the multiple component processes involved in intentional retrieval [10]. To this end, we manipulated encoding experience in Experiment 1 by means of a delayed match-to-sample test (Figure 1A) and then examined neural responses to subsequent, but task-irrelevant presentations of the stimuli (Figure 1B). We assessed blood-oxygen-level-dependent (BOLD) responses to paired repetitions of abstract shape items that had been held in working memory (WM) during an initial study phase and to paired repetitions of items that had been attended but not held in WM (hereafter referred to as the ‘Primed’ set of items). Critically, there was no requirement during the repetition of the stimuli to recall the information or the circumstances under which it had been encoded; a recognition test was later conducted outside the scanner to assess whether items committed to WM were associated with higher memory strength relative to the set of Primed items. Neural responses during trials with paired repetitions of WM and Primed items were measured relative to two additional conditions composed of trials with different items drawn from the WM and Primed sets (see Figure 1B and Methods); each item appeared twice over the course of a block of trials but never presented on the same trial. Reappearance of items across trials in these unpaired conditions therefore occurred with a longer inter-item lag than in the paired repetition condition where trials were composed of consecutive presentations of the same item. Note that RS is commonly assessed relative to a baseline of novel items. It is however well established that novel stimulus onsets can trigger neuronal enhancement in MTL regions [15]. Critically we aimed to assess the modulation of stimulus repetition effects by prior encoding experience (i.e. whether or not an item had been previously committed to WM) in the absence of novelty-related activation. We therefore took advantage of the frequently reported finding that neural repetition effects are attenuated as the inter-item lag increases [16], [17], [18], [19], [20], [21], [22], as are behavioural priming effects (for example [23]). A behavioural experiment indicated that repetition effects in response to our specially selected set of novel abstract shapes were greatly reduced with inter-item lag. For the purposes of this paper we will define neural repetition effects as the BOLD signal difference in response to short-lag, within trial paired repeats relative to unpaired conditions.

One possibility considered was that subsequent encounters with both WM and Primed items after the initial study phase would result in neural RS in MTL regions, consistent with a familiarity signal of prior occurrence [4], [5], [24]. Alternatively, variation in encoding experience may result in qualitatively different patterns of neural response to future encounters with stimuli. We additionally considered the possibility that repetition effects might be observed in regions outside the MTL, for example in regions of the prefrontal cortex which have also been shown to display variation in neural repetition effects in memory paradigms [25], [26], [27]. To anticipate the results, Experiment 1 showed that re-appearance of items that had not been associated with active encoding in WM resulted in consistent neural RS while encounters with items that had been held in WM did not. A post-scan recognition test confirmed that items from the WM set were recalled with greater accuracy than items from the Primed set; in Experiment 2, therefore, we considered the possibility that variation in the BOLD signal in response to previously studied stimuli might be associated with the strength of the memory trace. We employed the same procedure described above, but added multiple exposures of all critical items prior to the study phase in an effort to equate memory strength between the WM and Primed item sets. We questioned whether under these conditions the encoding experience following this pre-exposure phase would still have the power to modulate putative memory networks.

## Materials and Methods

### Ethics Statement

This research was approved by the Hammersmith and Queen Charlotte’s & Chelsea Research Ethics Committee, and all participants provided informed written consent.

### Behavioural Experiment

6 participants (4 male, age range: 21–36 years) took part. The 6 participants in this experiment did not take part in the subsequent fMRI studies (Experiments 1 and 2, described below). The experiment involved a brief recognition memory test which was carried out to establish whether behavioural repetition priming effects could be observed with the novel stimuli employed here. Participants were required to monitor a stream of abstract monochrome shapes presented for 1000 ms with a 500 ms inter-trial interval and indicate the presence of a repetition with a mouse click. 180 trials were performed, 60 of which featured a repetition of a previously presented shape. Repeated presentations of the stimuli were presented sequentially (lag 0, consonant with the paired repetition case) or were separated by 1 or 2 intervening trials (lag 1 and lag 2). Mean accuracy on the repetition detection task decreased significantly with increasing lag (F(2,10) = 26.058, p<.001) from 99% at lag 0 to 65% at lag 1 and 42% at lag 2. Accuracy at lag 1 and lag 2 did not differ significantly from chance (t(5) = 1.705, p>.05; t(5) = −1.147, p>.05). Mean reaction time increased linearly with increasing inter-item lag (F(2,10) = 13.983, p<.001) from 529.21 ms at lag 0 to 701.02 ms at lag 1 and 751.42 ms at lag 2. We conclude that clear differences in repetition priming effects between paired and unpaired repetitions can be observed with this set of novel stimuli and critically these priming effects are reduced as inter-item lag increases.

### Experiment 1

#### Participants

17 healthy participants (8 female) aged between 18 and 30 (mean = 22.63, SD = 2.55), with normal or corrected-to-normal vision, were recruited by means of an advertising campaign and were paid £20 for their participation. No participant reported prior history of neurological or neuropsychiatric disorders. Participants were all naïve with regard to experimental aims and hypothesis.

#### Experimental procedure

The experiment was performed in three stages; a memory study phase followed by a repetition phase was conducted within the MR environment. The experiment concluded with a recognition memory test performed outside the scanner. Figure 1 depicts sample trials from all experimental stages. The experimental tasks were programmed and presented with E-Prime v2.0 (Psychology Software Tools Inc., Pittsburgh, USA; www.pstnet.com/eprime.cfm).

Memory study phase: Participants’ experience with stimuli was manipulated by means of a delayed match-to-sample task (see Figure 1A). Each trial began with the word ‘Remember’ presented centrally on a black screen. A single stimulus was presented for 1000 ms, followed by a delay of 2000 ms. A probe item was then presented for 1000 ms, during which time participants were required to indicate by means of a button press whether the probe item matched the previously presented item (50% probability of match). There was an inter-trial interval of 1000 ms. Twenty abstract shapes, randomly selected, were presented as items to be remembered. Of these, 10 were presented in ‘match’ trials where the memory probe matched the cue item. These items were then discarded and were not re-presented in subsequent experimental stages. The remaining 10 shapes were presented as memory cues in ‘no-match’ trials where they were followed by a non-matching probe item. The shapes presented in the no-match trials were carried forward into subsequent experimental stages where the 10 memory cues formed the WM set and the subsequent 10 memory probes formed the Primed set. Memory cues and probe items were presented at central fixation for the same duration. The only difference between the WM and Primed sets was therefore the requirement to hold the stimuli in working memory over a delay period. The memory probes had to be processed and fully attended, but observers were not required to maintain an active memory trace. Thus the operational definition of encoding experience and the principal difference between WM and Primed items resided in the presence or absence of the requirement to commit the item to working memory.

Repetition phase: The repetition phase of the experiment consisted of four 30 second blocks. A single trial in all blocks consisted of two stimuli presented consecutively at fixation for 250 ms, separated by a 500 ms gap and followed by a 1500 ms intertrial interval. The four blocks differed in terms of the stimulus set displayed (WM or Primed) and the latency between stimulus repetitions. Each item from the relevant set was presented twice over the course of the block; within the paired repetition blocks, both presentations of an item occurred within the same trial while in the unpaired repetition blocks the two presentations of a given item occurred on different trials (see Figure 1B). We additionally ensured that, in the unpaired repetition blocks, the first item of a given trial did not match the last item of the previous trial. The lag between the first presentation and the second presentation ranged from 3.5–11 seconds with a mean of 6.2 seconds. The order of presentation of the items and the four block conditions (WM with paired repetition, WM with unpaired repetition, Primed with paired repetition, and Primed with unpaired repetition) was randomly selected for each participant.

In order to ensure that participants maintained attention on the display, they were instructed to monitor the central fixation point for a brief (100 ms) change in colour from white to red and to report this change by means of a button press. This change occurred on 20% of trials during the 1500 ms intertrial interval. The onset of these catch trials was distributed randomly within the repetition period across the different blocks. The entire experiment was performed twice within the same fMRI run using the same stimuli for the WM task and repetition phase as in the first round in order to maximise power. The composition of the WM and Primed sets did not vary between the rounds – each set was composed of the same 10 items in round 1 and round 2. Stimulus order and repetition block type were randomised in both runs.

Recognition memory phase: Following the completion of the experiment, participants performed an unexpected recognition test outside the MR scanner. Participants performed 30 trials. Items from the WM set, Primed set and 10 completely novel items were randomly selected. Participants were asked to indicate first whether the item was old or new and then to rate their confidence in that judgement on a scale from 1 (not very confident) to 3 (very confident). This is depicted in Figure 1C.

#### Statistical analysis

Behavioural data were analysed using repeated measures Analyses of Variance and paired sample t-tests with statistical significance defined as p<0.05, two-tailed.

#### Image acquisition/scanning parameters

MRI scanning was conducted using a Siemens Magnetom Verio 3T MRI scanner and a 32-channel head coil. Following a brief localizer scan to determine the orientation of the subject’s head within the field, 176 T1 weighted anatomical sagittal images were acquired with an FOV of 220×220 mm, TR of 1900 ms, TE of 2.48 ms and slice thickness of 1 mm, leading to a voxel resolution of 1×1×1 mm. A single functional run was then conducted during which a T2* weighted echo planar imaging sequence was used to obtain 38 contiguous sagittal slices covering the whole brain. 420 volumes were acquired with an FOV of 222×222 mm, TR of 2200 ms, TE of 30 ms and slice thickness of 3 mm. The resulting voxel resolution was 2.4×2.4×3.0 mm.

#### Imaging data analysis

fMRI data processing was carried out using FEAT (fMRI Expert Analysis Tool) Version 5.98, part of FSL (FMRIB’s Software Library, www.fmrib.ox.ac.uk/fsl). The first 6 volumes of the EPI scan were removed from the dataset, leaving 414 scans. The following pre-statistics processing was applied: motion correction using MCFLIRT [28]; non-brain removal using BET [29]; spatial smoothing using a Gaussian kernel of FWHM 7.0 mm; high-pass temporal filtering (Gaussian-weighted least-squares straight line fitting, with sigma = 50.0 s). Time-series statistical analysis was carried out using FILM (FMRIB’s Improved Linear Model) with local autocorrelation correction [30].

Statistical analysis was performed by modelling paired repetition versus unpaired repetition and working memory versus priming conditions (boxcar functions convolved with the hemodynamic response function) as explanatory variables within the context of the general linear model on a voxel-by-voxel basis. As trial-by-trial information during the assessment of the neural repetition effects was not required in this experiment a block design was used to maximise design efficiency [31] and increase power to detect potentially small differences in paired-unpaired effects between the WM and Primed conditions. Additional explanatory variables (cue onsets in both match and mismatch trials in the working memory task; dummy task onsets in the repetition phase; errors; motion realignment parameters) were included as regressors of no interest. The temporal derivative of the haemodynamic response function was also added to the model for each explanatory variable. Z (Gaussianed T/F) statistic images were thresholded using clusters determined by a voxelwise Z threshold of 2.3 and a (corrected) cluster significance threshold of p = 0.05 [32], unless otherwise noted. Registration to high resolution structural images of each individual subject was carried out using FLIRT [28], [33] and all high-resolution structural images were co-registered to standard (Montreal Neurological Institute) space. Higher-level analysis was carried out using FLAME (FMRIB’s Local Analysis of Mixed Effects) stage 1 [34], [35], [36]. Z statistic images were created for the four conditions (WM-paired, WM-unpaired, Primed-paired, Primed-unpaired) and thresholded in the same manner as above.

### Experiment 2

#### Participants

19 healthy participants (8 female) aged between 18 and 30 who had not participated in Experiment 1 were recruited for Experiment 2. The same exclusion criteria as in Experiment 1 were applied.

#### Experimental procedure

The memory study, repetition and recognition test phases were performed as described in Experiment 1. Prior to the start of the first memory study block, a Pre-Exposure phase was performed, in which the 10 WM and 10 Primed shapes were presented 10 times each in random order, for 1 second and with a 100 ms interval. Participants were instructed to monitor the display for a repetition of any shape, in which case they were to press a button. 10 additional shapes were included in this stage of the experiment for this purpose; on 50 of the 300 trials pre-exposure trials, one of these additional shapes was presented twice in a row. None of these shapes were presented again during the rest of the experiment, and the WM and Primed shapes did not repeat in this fashion during the pre-exposure phase.

#### Imaging acquisition and analysis

fMRI acquisition was carried out in the same manner as in Experiment 1, with the single EPI run extended to 588 volumes to allow for the addition of the pre-exposure phase. Statistical analysis was conducted as before, by modelling paired repetition versus unpaired repetition and working memory versus priming conditions, resulting in thresholded Z statistic images for the four conditions (WM-paired, WM-unpaired, Primed-paired, Primed-unpaired). New regressors of no interest modelled stimulus onsets, repeat trials and errors of omission during the pre-exposure phase.

## Results

### Experiment 1: Effect of Encoding Experience during a Study Phase on Future Neural Re-appearance Effects

#### Working memory task

WM performance in the initial study phase (Figure 1A) was very high (mean accuracy = 95%; SD = 3.1%), indicating that items assigned to the WM set were successfully maintained in WM.

#### Target detection during repetition

In order to ensure attention to the visual display during the repetition phase (Figure 1B), we asked participants to monitor the white fixation cross for a colour change and to respond to any change by means of a button press. Performance on these catch trials was high (mean detection accuracy = 91%; SD = 8%) and did not differ significantly across the four repetition blocks.

#### fMRI results

**Figure 2:**
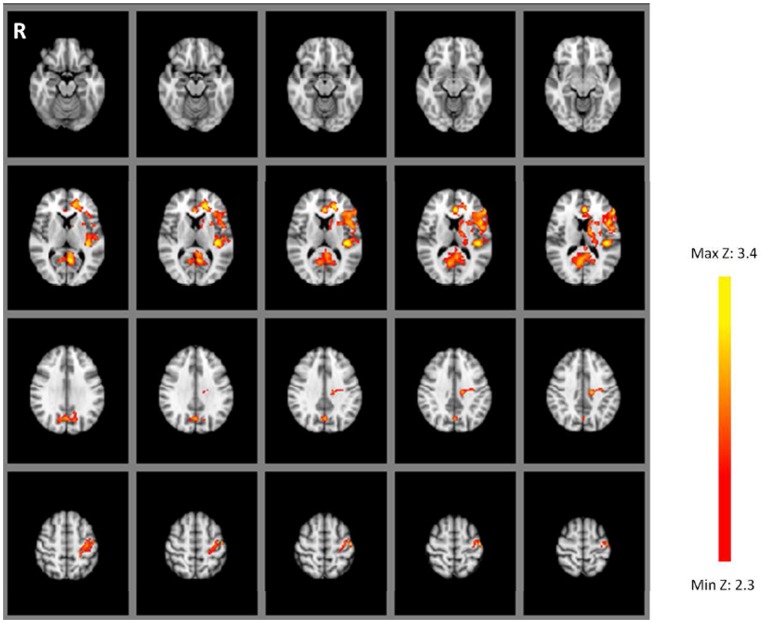
Data from Experiment 1. Percentage signal change in paired repeats relative to the unpaired baseline. Regions showing repetition suppression in both WM and Primed conditions. Voxelwise threshold: Z = 2.3, p<.05, corrected for multiple comparisons. Images are in radiological orientation (right = left).

Whole brain analyses were conducted with the goal of assessing regions displaying distinct neural repetition effects in the WM and Primed conditions. We first assessed main effects of memory condition. No significant differences in BOLD response were observed in either direction (i.e. WM >Primed or Primed > WM), using contrasts specified as [+1+1−1−1] and [−1−1+1+1] respectively implemented as t-tests. We next tested for a main effect of repetition by contrasting performance in the paired repetition case against the unpaired baseline. While no voxels showed significant RE (i.e. increased activity in the paired repetition condition relative to the unpaired baseline) across both WM and Primed conditions, a common pattern of neural RS (i.e. reduction of activity in the paired repetition condition relative to the unpaired baseline) was observed in both WM and Primed conditions following specification of the contrast [−1+1−1+1]. As displayed in Figure 2 and Table 1, this pattern of response was observed in a network of left-lateralised regions including the inferior frontal gyrus, anterior and posterior cingulate and also in the precuneus, which has been shown to display RS during object priming and recognition memory processes [37], [38].

**Figure 3:**
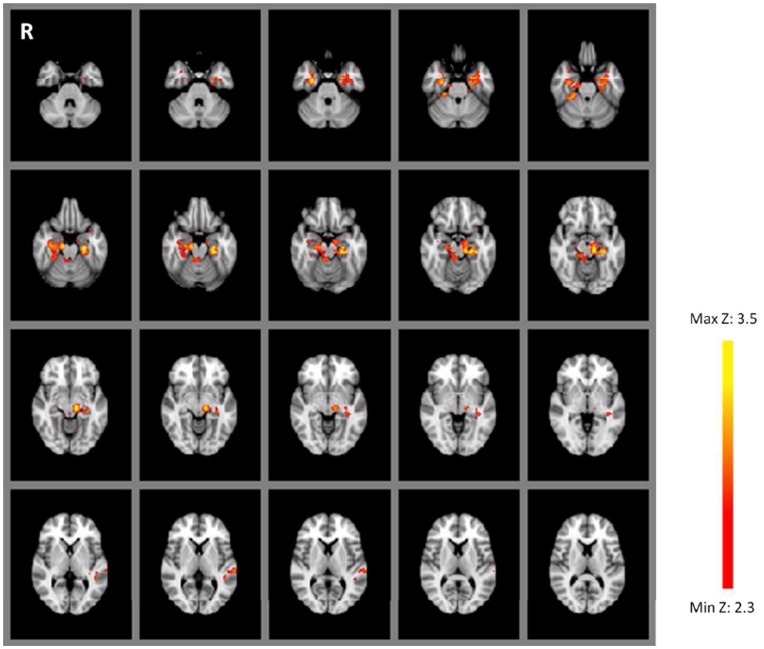
Data from Experiment 1. Percentage signal change in paired repeats relative to the unpaired baseline. Regions displaying attenuation of neural activity in the paired repetition condition relative to baseline during the Primed condition but not the WM condition. Z = 2.3, p<.05, corrected for multiple comparisons. Images are in radiological orientation (right = left).

**Figure 4:**
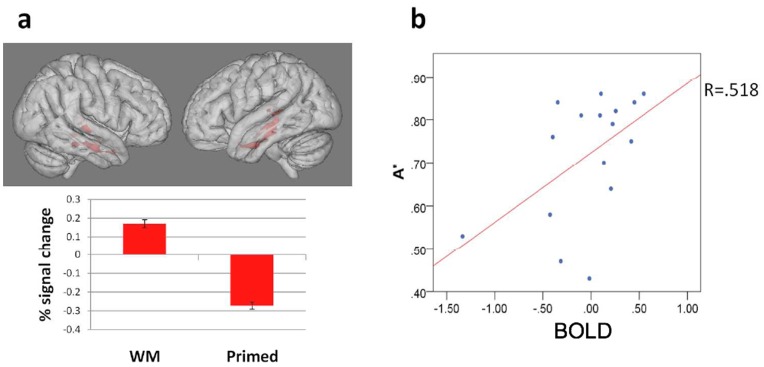
Data from Experiment 1. (A) Percentage signal change in MTL (paired repeats relative to the unpaired baseline) in WM and Primed conditions. Z = 2.3, p<.05, corrected for multiple comparisons. (B) Scatterplot of relationship between BOLD signal during WM paired repetition blocks and memory sensitivity during WM item trials in the subsequent recognition test.

Our main goal was to look for regions displaying variation in neural repetition effects as a function of encoding context. To this end, we applied an interaction contrast implemented as a t-test which may be written as, for example, WM_paired_ > Wm_unpaired_ & Primed_paired_ < Primed_unpaired_, leading to a contrast specification of [1−1−1 1]; we also tested the opposite interaction contrast [−1 1 1−1]. These interaction contrasts are critical for the aims of this study as they directly compare of the effect of the prior encoding experience (e.g. WM vs. Primed) on the paired/unpaired effect. Whole brain analysis utilising the former interaction contrast revealed bilateral clusters in the MTL covering the hippocampus, parahippocampal gyrus and partially extending into the fusiform gyrus and left amygdala (see Figure 3 and Table 2). As Figure 4a shows, RS (i.e. reduced BOLD signal in the paired case relative to unpaired case) was observed in the Primed condition but not in the WM condition, *post-hoc* t-tests on individual subject beta values from the MTL cluster show that the difference between paired and unpaired repetition was statistically significant in the Primed case (t(16) = −3.858, p<.001) but not in the WM case (t(16) = 1.207, p>.05). There were no significant effects of the opposite interaction contrast (i.e. [−1 1 1−1]). We also note that the expression of neural repetition effects as a function of the memory set (WM vs. Primed) did not differ between the two scanning runs (see Methods).

**Table 1:** Regions showing repetition suppression in both WM and Primed conditions (experiment 1).

Region	Brodmann Areas	Hemisphere	Z	Peak MNI coordinates	Size (voxels)
Inferior frontal gyrus, anterior cingulate, paracingulate gyrus	24,32,45,48	Left	3.72	−42 –24 16	2997
Precuneus, posterior cingulate	17,18,23,29	Bilateral	3.79	−2 –50 2	2323
Precentral gyrus, postcentral gyrus, central sulcus, parietal operculum	2,3,4,31,40	Left	3.75	−36 –18 48	870

**Table 2:** Regions displaying a reduced BOLD signal relative to baseline during the Primed condition but not during the WM condition. (Experiment 1).

Region	Brodmann Areas	Hemisphere	Z	Peak MNI coordinates	Size (voxels)
Medial temporal lobe: hippocampus, parahippocampal gyrus, amygdala, fusiform gyrus	20,21,27,28, 30,36	Left	3.81	−12 –24 –12	1224
Medial temporal lobe: hippocampus, parahippocampal gyrus, fusiform gyrus	20,28,35,36, 37	Right	3.55	16 –18 22	873

#### Recognition test

Following the completion of the WM and repetition phases of the experiment, participants received an unexpected memory test. This test is depicted in Figure 1C and was performed outside the scanner. Due to a technical problem data from this test were not collected from the first participant. Signal detection theory was used to calculate a measure of memory sensitivity (A’). Hits (i.e. responding ‘old’ to the presentation of an old item) were calculated separately for WM and Primed item sets. A common rate of false alarms (‘old’ responses to new items) was calculated during presentation of novel items. Sensitivity in the WM condition (mean A’ = 0.72, SD = 0.14) was significantly higher than that in the Primed condition (mean A’ = 0.62, SD = 0.22); t(16) = 2.199, p<.05 (see Table 3). A significant difference in confidence ratings among the three conditions (WM, Primed, Novel) was also detected (F(2,30) = 9.8, p<.001). Planned post-hoc contrasts indicated that confidence ratings in the WM condition were higher than in the Primed (F(1,15) = 10.895, p = .005) and Novel (F(1,15) = 21.823, p = .000) conditions, which did not differ from one another (F(1,15) = 0.396, p = .538). Mean response time on the recognition test did not differ among the three conditions (F(2, 30) = 1.941, p = .16).

**Table 3:** Sensitivity (A’) and confidence ratings from Experiment 1 and Experiment 2.

		*A’ (SD)*		*Confidence rating (SD)*		*N*
**Experiment 1**						
WM	.7224 (.14)		2.36 (.42)		17	
Primed	.6153 (.22)		2.12 (.44)		17	
Novel	–		2.07 (.35)		17	
**Experiment 2**						
WM	.8794 (.11)		2.51 (.29)		19	
Primed	.8137 (.22)		2.35 (.35)		19	
Novel	–		2.27 (.33)		19	

#### Relationship between BOLD responses and memory performance

Finally, we looked for a relationship between the neural responses in the MTL and behaviour. We hypothesised that BOLD signal intensity during the repetition blocks would be predicted by subsequent performance in the surprise recognition test performed outside the scanner. Separate analyses were conducted for the WM and Primed conditions; memory sensitivity (A’) and confidence ratings for each subject were entered as predictor variables into a backwards stepwise regression where the dependent variable was BOLD signal during the repetition blocks extracted from a mask of the MTL regions that displayed RE during the WM condition and RS during the Primed condition. BOLD response during the WM repetition blocks was found to be significantly predicted by A’ in WM recognition trials (F = 5.132, p = .04; R^2^ = 0.268; see Figure 4b), indicating that memory sensitivity accounted for 27% of the variance in BOLD response. In other words, participants with greater response to incidental repetitions of items that had been previously held in WM tended to show higher memory sensitivity for those items during the subsequent recognition test. Confidence ratings did not significantly predict BOLD response, and their inclusion in the regression model did not improve the predictive power of the regression model (F = 2.625, p = .11; R^2^, = .178). Memory sensitivity and confidence ratings in response to Primed stimuli in the recognition test did not predict BOLD response during the Primed repetition blocks (F = .465, p = .638).

### Experiment 2: Influence of Pre-exposure of Stimuli

#### Working memory task

Mean accuracy in the study phase WM task was 89% with a standard deviation of 10%.

#### Target detection during repetition

Mean performance on repetition block catch trials was very accurate (mean = 96%, SD = 11%) and did not differ across the 4 repetition blocks (F(3,27) = .895, p>.05).

#### fMRI results

**Figure 5:**
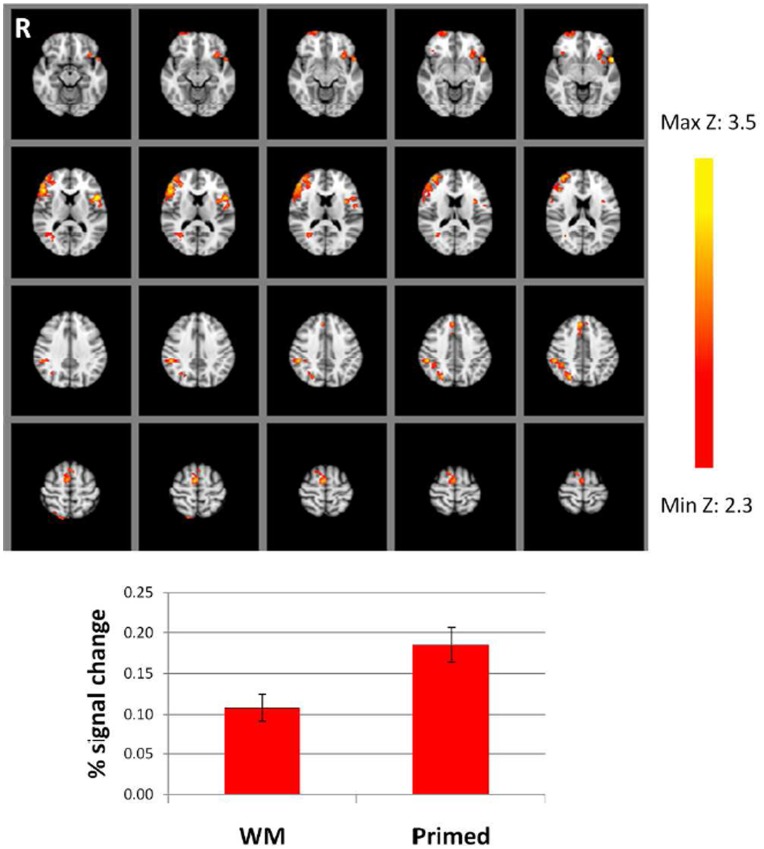
Data from Experiment 2. Percentage signal change in paired repeats relative to the unpaired baseline. Regions showing repetition enhancement common to both WM and Primed conditions. Z = 2.3, p<.05, corrected for multiple comparisons. Images are in radiological orientation (right = left).

No main effects of memory condition (WM > Primed or Primed > WM) were observed. A main effect of repetition condition (paired repetition > unpaired repetition), common to both WM and Primed sets, was observed in bilateral inferior frontal gyrus (IFG) and right posterior parietal cortex (PPC; see Figure 5 and Table 4). We lowered the voxelwise threshold to Z = 1.7, while still correcting for multiple comparisons at p<.05, in order to look for activation in MTL regions. A small cluster of significantly activated voxels was now observed in the left hippocampus (MNI coordinates: −28−14−14). No regions showed a pattern of RS common to both memory conditions.

**Table 4:** Regions displaying greater activation during repetition than baseline conditions (RE).

Region	Brodmann areas	Hemisphere	Z	Peak MNI coordinates	Size (voxels)
Lateral occipital cortex, supramarginalgyrus, superior parietal lobule	7,40,19	Right	3.63	18 –82 54	1405
Inferior frontal gyrus, middle frontal gyrus, precentral gyrus	43,44,45,48	Right	3.67	48 26 10	1112
Inferior frontal gyrus, orbitofrontal cortex, precentral gyrus, central opercular cortex	44,47,48	Left	4.12	−48 14 4	939
Superior frontal gyrus, supplementarymotor cortex, paracingulate gyrus	6,8,32	Right	3.44	6 38 42	815
Frontal pole	10,11,46	Right	3.6	34 56 18	797

Z = 2.3, corrected for multiple comparisons (experiment 2).

We also tested for differences in neural repetition effects between the WM and Primed conditions, but no significant effects were observed in either direction (i.e. reduced RS in WM condition relative to Primed condition: contrast specification [+1−1−1+1]; increased RS in WM condition relative to Primed condition: contrast specification [−1+1+1−1]. A mask of the bilateral MTL regions activated during Experiment 1 (see Figure 2) was applied and a small volume correction used to search for sub-threshold effects in this region. No other significant effects were observed in this region of interest. Thus, in contrast to Experiment 1, here we observed that following massive exposure of the items the expression of neural repetition effects was not modulated by whether or not the items had been held in WM during the prior study phase.

#### Recognition test outside the scanner

A paired samples t-test confirmed that mean A’ did not differ between the WM and Primed conditions (t(18) = 1.649, p>.05), suggesting that memory strength was equated amongst WM and Primed items. A repeated measures ANOVA of confidence ratings indicated a significant difference in confidence across the 3 conditions (F(2, 36) = 3.606, p<.05). Planned contrasts indicated that confidence ratings were significantly higher in response to WM set items than Novel items (t(18) = 2.683, p<.05), although there was no significant difference in confidence between items from the Primed and Novel sets (t(18) = −76, p>.05). A marginally significant difference was observed between the WM and Primed conditions (t(18) = 2.104, p = .05) such that confidence ratings were slightly higher in response to WM items.

#### Comparison with experiment 1

Independent-samples t-tests indicated that memory sensitivity, indexed by A’, was higher in Experiment 2 following pre-exposure than in Experiment 1 in both the WM (t(34) = 3.792, p<.001) and Primed (t(34) = 2.691, p<.05) conditions. Confidence ratings in the WM, Primed and Novel conditions did not differ between Experiment 1 and Experiment 2 (p>0.05).

Taken together, these results indicate that the strength of the memory trace increased following massive exposure of the stimuli in Experiment 2 relative to Experiment 1. Further, while memory sensitivity for WM items in Experiment 1 was higher relative to the Primed set, this no longer appeared to be the case in Experiment 2.

## Discussion

Experiment 1 showed that neural repetition effects in the MTL, including the hippocampus and parahippocampal gyrus, were dependent on the nature of encoding experience with items during the earlier study phase; RS, which has been conceptually linked with priming mechanisms [2] was observed after repetition of items which had previously been attended. No evidence for neural RS was observed following repetition of items which had to be actively rehearsed in WM. Rather, a trend towards the opposite pattern – neural RE – was observed in this condition, but did not reach statistical significance. Interestingly, participants with a higher degree of neural RE in the MTL complex displayed higher memory sensitivity in a later, surprise recognition test.

Reduction and enhancement of activity in the MTL with stimulus repetition has been observed in memory paradigms that required observers to make intentional retrieval-based responses in recognition tasks [4], [5], [6], [7], [8], [9]. Further, neural repetition effects in MTL and inferior temporal regions are known to be modulated by task requirements, for example, by the need to respond to the presence of an item that matches the contents of WM [39], [40], [41]. Critically, no task during the assessment of the neural repetition effects in the present study required intentional orientation to retrieve the previously experienced information, or encouraged discrimination between the two classes of stimuli or the context in which they were encoded.

It could be argued that neural repetition effects in the current study were driven by perceptual expectation. Because we used a blocked design the occurrence of paired stimulus repetition could be anticipated. While there is some evidence to suggest that neural RS may be enhanced by expectation [42] recent work indicates that neural RS effects may remain even when perceptual expectation effects are controlled for by diverting attention away from the stimuli [43], suggesting that perceptual expectation does not account for the entirety of neural RS. More importantly, any effect of expectation in the current study should have been present both in the WM and Primed conditions of Experiment 1 and 2, yet Experiment 1 showed neural RS in the Primed case only while the (opposite) trend towards neural RE was observed in the WM case. Experiment 2 showed no hint of neural RS even though expectation effects could presumably be at work. Taken together, this pattern of results is difficult to explain in terms of expectation-based accounts. Instead we provide an account below linking neural repetition effects with the strength of the memory trace.

The surprise recognition test performed outside the scanner suggests that items from the WM set in Experiment 1 were associated with a stronger memory trace relative to the Primed set and the magnitude of MTL responses across individuals to repetition of WM items predicted subsequent recognition performance. The same relationship was not found between neural response and recognition memory performance in the Primed condition. Interestingly, when the memory strength of items from both WM and Primed sets was equated by the use of massive pre-exposure prior to the study phase (in Experiment 2), a common pattern of neural RE in both WM and Primed conditions was observed. Although neural RE was detected in the left hippocampus in Experiment 2, the presence of RE extended to putative neocortical substrates of explicit control of retrieval including the IFG [44], [45] and the PPC [46], [47]. Notably, no brain regions displayed RS following massive exposure of the items. Taken together these findings suggest that the general expression of neural RE is a consequence of the effect of encoding on the strength of the memory trace. This suggestion appears in contrast with a body of research suggesting that repeated presentations of stimuli result in a reduction, or adaptation, of the neural response [48], [49]. It is interesting to note that decreases in brain activity, namely increases in adaptation effects or neural RS, with increasing number of repetitions in the aforementioned studies was typically found in regions that implement visual perceptual processing (e.g. the lateral occipital cortex), while our finding of decreased RS and expression of RE was observed in regions that implement higher-level processing such has the MTL complex, IFG and the PPC. Thus we suggest that experience-dependent neural responses may be implemented differently across different functional brain regions. Moreover there is evidence that experience-dependent plasticity in early visual regions (e.g. shape perceptual learning though massed practice) may lead to neural RE effects [50] which is consistent with what we found. There may be a tipping point based on the amount of exposure to the information whereby adaptation effects, or neural RS, turn into the expression of neural RE. Our pattern of results supports this view.

The reduced engagement of the hippocampus during Experiment 2 relative to Experiment 1 might seem contradictory at first, given the proposed linear relationship between hippocampal activity and memory strength [51], [52] (though see [53]) and given that memory strength - indexed by performance in the recognition tests - was higher in Experiment 2 than in Experiment 1. Note, however, that activation in the hippocampus in response to complex stimuli has been shown to decrease linearly with increasing exposure [53], [54]. It is therefore possible that the massive exposure of both item sets may have fundamentally altered the manner in which stimuli are represented in the hippocampus and surrounding MTL regions. Below we provide an account of how this pattern of neural activity could arise.

Pattern completion is the process by which the hippocampus extrapolates from a single or partial stimulus to retrieve a complete representation. This is the mechanism whereby the presentation of a single feature (e.g. mouth), which may have never before been seen in isolation, can reactivate the neural representation of the whole object (face). Pattern completion is supplemented by pattern separation, whereby discriminable stimuli result in distinct hippocampal representations. Recent work with high-resolution fMRI has demonstrated that the manifestation of pattern completion and pattern separation in the human hippocampus is influenced by the perceived similarity between stimuli [55] whereby high similarity between items can cause hippocampal pattern separation to fail [56]. The distinctiveness of the stimuli in the present study increased following massive exposure in Experiment 2, as evidenced by the enhanced memory performance relative to Experiment 1. This may have tipped the balance in favour of pattern separation rather than completion as, following massive exposure of the items, the prior encoding context (WM vs. Primed) may no longer have acted as a contextual cue to ‘group’ the items. We suggest that massive exposure of the items in Experiment 2 may have promoted the occurrence of pattern separation and the creation of more distinct neural representations for each item. Under these conditions, any neural repetition effect ought to become more item-specific, resulting in reduced overlap between the neural repetition responses within each WM/Primed item set. Thus the decreased probability of pattern completion based on encoding context may explain the apparently reduced engagement of the hippocampus and the MTL in Experiment 2. This coincides with the suggestion by Raposo *et al.* [57] that neuronal enhancement in response to repetition of semantically primed words reflects fine-grained distinctions in the representation of highly similar stimuli. Future research using high-resolution imaging methods would be useful to establish the influence of encoding experience on pattern completion and separation processes in the hippocampus and in other regions of the MTL.

Moving beyond the MTL, the results of Experiment 2 showed large neural RE effects in a number of memory-related regions including the IFG [44], [45], [58] and the PPC [46], [47] (see below for discussion). The left IFG has been involved in explicit control of episodic retrieval [45], namely, resolving competition from multiple stimuli [25] and also shows variation in neural repetition effects by high-level object and semantic priming [26], [27]. To account for the dissociation between the overall neural RE effects found in these neocortical regions following massive pre-exposure and the predominant role of the MTL in Experiment 1, it is worth considering the following: from a neurophysiological perspective, it is known that while synaptic connections in the MTL are extremely labile, with long-term potentiation often observed after very brief periods of stimulation, much longer and more intense periods of stimulation are required to produce synaptic plasticity in higher cortical regions [59], [60]. The few occurrences of each stimulus in Experiment 1 may have been insufficient to enact the changes observed in the neocortex following heavy pre-exposure of the stimuli in Experiment 2.

Left prefrontal regions, in particular the left IFG, have been implicated in ‘source memory’, the recollection of contextual associations between items during encoding [61], [62]. The PPC has also been associated with memory retrieval in recent years, with dorsal PPC involved in the allocation of top-down attention to memory in the service of retrieval [47], [63] while the more ventral PPC has been linked to recollection processes [6], [46], [58], [64]. Our PPC findings are supported by recent studies where response in ventral PPC has been associated with the amount of information retrieved even when this is not the basis of a recognition decision [65]. A similar result in the PPC has been shown to be dependent on whether the mnemonic status of the stimulus is associated with current goals [66]. Our study however demonstrates that putative substrates of memory, including the PPC, and also the MTL, may be engaged by the re-occurrence of a prior event in the absence of memory goals, highlighting the role of encoding experience in determining the manner in which future encounters with old information are represented in the brain.
